# Effect of orthostatic hypotension on long-term prognosis of elderly patients with stable coronary artery disease: a retrospective cohort study

**DOI:** 10.3389/fcvm.2024.1342379

**Published:** 2024-04-12

**Authors:** Jiaman Hu, Jianing Chi, Hua Cai, Ningxia Wu, Pengfei Li, Yuekang Huang, Cailong Lin, Yingying Lai, Jianyu Huang, Weihua Li, Peng Su, Min Li, Zhongqiu Lin, Lin Xu

**Affiliations:** ^1^ Department of Geriatric Cardiology & Branch of National Clinical Research Center for Geriatric Diseases & Guangzhou Key Laboratory of Cardiac Rehabilitation, General Hospital of Southern Theater Command, Guangzhou, China; ^2^ School of Public Health, Guangdong Pharmaceutical University, Guangzhou, China; ^3^ The First School of Clinical Medicine, Southern Medical University, Guangzhou, China; ^4^ Graduate School, Guangzhou University of Chinese Medicine, Guangzhou, China; ^5^ The Sixth Affiliated Hospital, School of Medicine, South China University of Technology, Foshan, China

**Keywords:** stable coronary artery disease, orthostatic hypotension, all-cause mortality, cardiovascular disease mortality, prognosis, cohort study

## Abstract

**Background:**

The long-term prognosis of patients with stable coronary artery disease (CAD) combined with orthostatic hypotension (OH) has rarely been reported. This research was designed to examine whether OH increases the risk of all-cause mortality and cardiovascular death among patients with stable CAD.

**Methods:**

We retrospectively analyzed retired military personnel over 65 years of age who were hospitalized at the General Hospital of Southern Theater Command of the Chinese People’s Liberation Army between March and July 2010. A total of 924 patients with stable CAD were included, among whom 263 had OH. The risk of all-cause mortality and cardiovascular death in OH and non-OH groups were analyzed with the Cox proportional hazards models, and restricted cubic spline plots were utilized for subgroup analyses. Furthermore, competing risk models were applied for sensitivity analyses.

**Results:**

The median age of the patients was 82.00 (80.00–85.00) years. Over 159 months of follow-up, the loss to follow-up rate was 2.27%, and all-cause mortality was observed in 574 (63.57%) patients, including 184 with OH. Moreover, cardiovascular death occurred in 127 patients (13.73%), with 58 cases associated with OH. Although the relationship between OH and all-cause mortality was non-significant [body mass index (BMI) < 25 group, adjusted hazard ratio (HR) = 1.10 with a 95% confidence interval (CI): 0.82–1.40; BMI ≥ 25 group, adjusted HR = 1.30, 95% CI: 0.98–1.70], it was independently related to a growing risk of cardiovascular death (adjusted HR = 1.80, 95% CI: 1.20–2.60). This finding was further validated by using a competing risk model (subdistribution HR = 1.74, 95% CI: 1.22–2.49). Moreover, age, low-density lipoprotein cholesterol, and frequency of hospital admissions were identified as risk factors of cardiovascular death among patients with OH (*P* < 0.05).

**Conclusion:**

Our study, based on retired military personnel with stable CAD, found that OH led to a significantly higher risk of cardiovascular death, but it was not noticeably associated with all-cause mortality on long-term prognosis.

## 1 Introduction

Coronary artery disease (CAD), one of the most common cardiovascular diseases (CVDs), constitutes a substantial burden on the global health. CAD mainly causes the depletion of disability-adjusted life years (DALYs) across the world. Low- and middle-income states disproportionately share this burden, occupying almost 7 million fatalities and 129 million DALYs per year (1, 2). Moreover, the annual death rate attributed to CAD decreased by 19.2%, while the actual number of deaths increased by 0.9% from 2010 to 2020 (3). In addition, the morbidity and mortality due to CAD continue to rise as society ages. Several studies have shown that the risk of death from CAD in the elderly is associated with autonomic dysfunction (4).

Orthostatic hypotension (OH) is acknowledged as a manifestation of autonomic dysfunction (5). OH often occurs when a person transitions from lying or sitting to standing, resulting in a substantial drop of blood pressure (BP). This can lead to various symptoms, including dizziness, light-headedness, and, in severe cases, fainting or falling (6–8). It is highly prevalent in the over-65 population, influencing one in five community-dwelling elderly people and almost one in four elderly people in long-time care (9). Meanwhile, OH can cause large fluctuations in blood pressure, which leads to inadequate blood supply to the brain and heart, resulting in an increased risk of cardiovascular events (10). In a meta-analysis (11) including a total of four studies on outcomes related to OH and CAD, only two studies have displayed a close connection between OH and CAD, while the other two have not. More evidence and arguments are required to establish a causal association between OH and the incidence risk of CAD-related events. In addition, there is a lack of studies on stable CAD populations to examine whether OH increases the risk of all-cause mortality and cardiovascular disease-related mortality in patients.

This research, aiming to explore the relationship between all-cause mortality and cardiovascular disease-associated mortality in stable CAD patients, is the first cohort study in China dedicated to examining the long-term prognosis of patients with stable CAD combined with OH.

## 2 Methods

### 2.1 Research design and population

**Figure 1:**
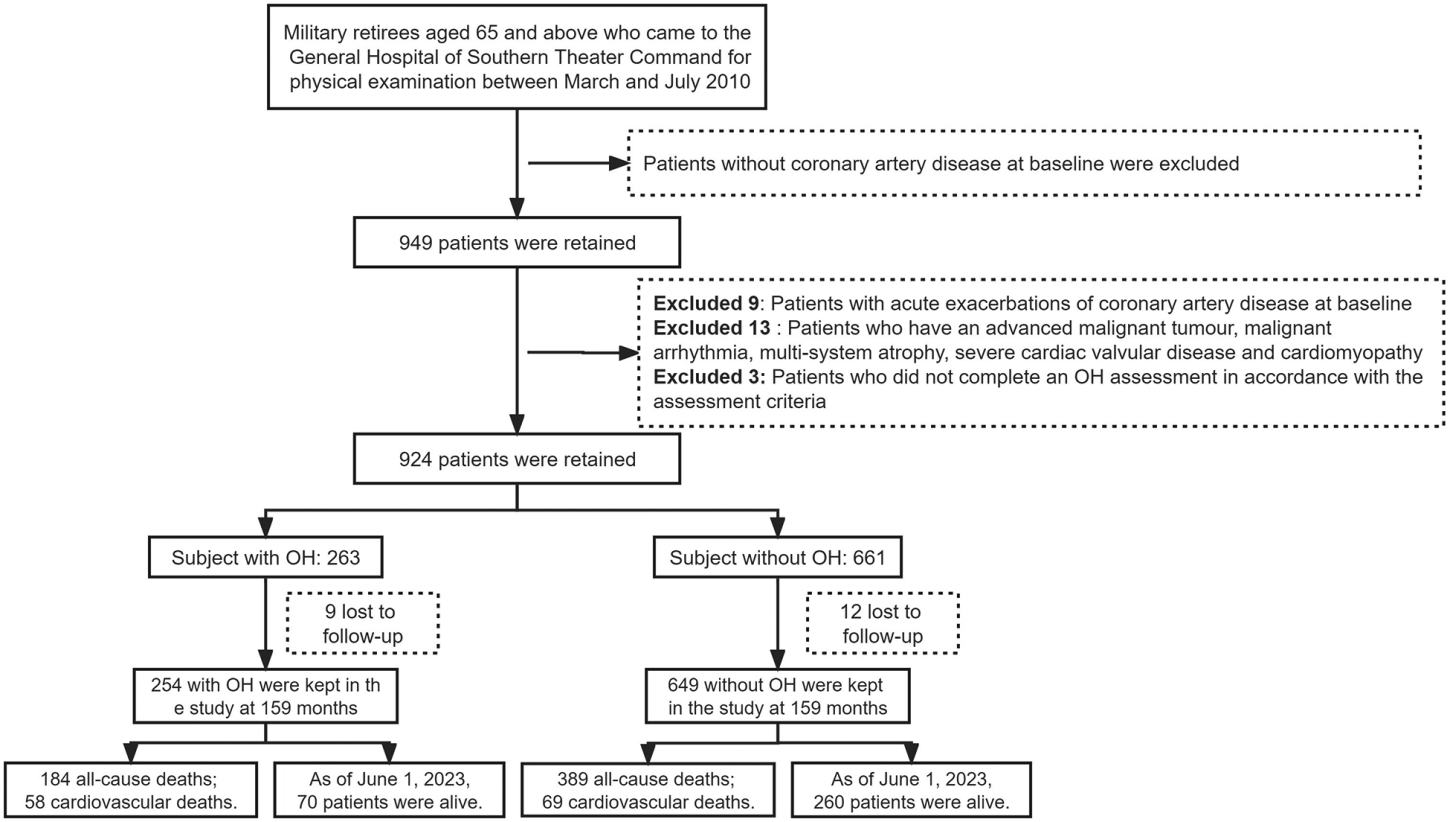
Flowchart of the data collection process for the research.

The research data were obtained from physical examination conducted between March and July 2010 of retired military personnel aged 65 years or older at the General Hospital of Southern Theater Command. We employed a retrospective cohort study design and identified a total of 924 eligible patients with baseline data. Among these, the case group (i.e., OH group) consisted of 263 patients presenting both CAD and OH, while the control group (i.e., non-OH group) comprised 661 patients with CAD. Figure 1 illustrates the data collection workflow for this study. This prognostic research was approved by the Ethics Committee of the General Hospital of Southern Theater Command (Project Ethics No.: NZLLKZ2023056).

The inclusion criteria were as follows: (1) patients diagnosed with chronic CAD; (2) patients were able to be active in standing; and (3) age ≥65 years. The diagnostic criteria for CAD were based on the “2023 AHA/ACC/ACCP/ASPC/NLA/PCNA Guideline for the Management of Patients with Chronic Coronary Artery Disease” (12).

The exclusion criteria were as follows: (1) patients without CAD at baseline; (2) patients with acute exacerbation of CAD; (3) patients with advanced malignant tumors, malignant arrhythmias, multiple system atrophy, severe heart valve disease, or acute cardiomyopathy at baseline; (4) patients who did not complete the OH assessment according to assessment criteria; and (5) individuals lacking data on relevant covariates at baseline or those who were lost to follow-up during observation.

### 2.2 OH diagnostic methods

The average blood pressure measurements for the participants were taken in both lying down and standing up positions during the physical examination, conducted by qualified medical personnel using active standing test (13). Before the measurement, the participants were instructed to have a rest for at least 5 min, refrain from caffeine and smoking, and empty their bladders thirty minutes beforehand. To prevent confusion with postprandial hypotension, the test was typically conducted two hours after a meal (14, 15). Sitting and lying blood pressures were sequentially measured three times at 1–2 min intervals, meanwhile, and sitting heart rate (HR) was documented. Orthostatic hypotension was described as a decrease of ≥20 mmHg in systolic blood pressure (SBP) and/or ≥10 mmHg in diastolic blood pressure (DBP). OH was diagnosed in all cases that met either of these conditions (16, 17). The variables OH_0_ and OH_2_ referred to orthostatic hypotension assessed immediately and 2 min after standing upright, respectively.

### 2.3 Outcomes

The main endpoints of the research were shown as all-cause mortality and cardiovascular death. To diagnose patient outcome events, two doctors separately coded all reported events. In cases of controversy, resolution decisions were reached through consultation with the third medical expert. Death codes for cardiovascular diseases were assigned on the basis of the International Classification of Diseases, Tenth Revision (ICD-10). The ICD-10 codes adopted for coding were G45, I01, I03-182, I87, I95-I99, F01, Q20–Q28, and R96.

### 2.4 Other risk factors

The hospital physical examination center provided data on the age, sex, smoking, alcohol consumption, physical activity, HR, SBP, DBP, pulse pressure (PP), fasting blood glucose (FBG), creatinine, blood urea nitrogen (BUN), uric acid (UA), total cholesterol (TCHO), high-density lipoprotein cholesterol (HDL-C), low-density lipoprotein cholesterol (LDL-C), triglyceride (TG), and estimated glomerular filtration rate (eGFR). The histories of anti-hypertensive and hypoglycemic medication usage, hypertension, diabetes mellitus (DM), heart failure (HF), myocardial infarction (MI), and stroke were self-reported. The calculation of body mass index (BMI) was on the basis of weight and height. The measurements of waist and hip circumferences were made for the waist-to-hip ratio (WHR).

### 2.5 Statistical analysis

The missing data were filled in using multiple interpolation. Continuous variables were shown as either mean ± standard deviation (SD) for normal distribution data or median combined with interquartile range (IQR) for abnormal distribution data. Statistical comparisons were made with independent Student's *t*-tests for normal distribution data and the Mann–Whitney *U* test for abnormal distribution data. The comparison of categorical variables shown as counts and percentages was made with the Chi square test or Fisher’s exact test. The cumulative rates of occurrences over time and the median survival time were estimated with the Kaplan–Meier survival curves and the assessment of group diversities was made with the log-rank test.

Proportional Hazard (PH) presumptive tests were determined based on statistical tests and Schoenfeld's residual plots, with the analysis stratified for variables that did not satisfy the PH test. The calculation of hazard ratios (HR) and 95% confidence intervals (CI) was made for every effector using a one-way Cox proportional hazards model. The differences in risk of all-cause mortality and cardiovascular disease mortality between OH and non-OH were assessed using adjusted Cox regression models, taking into account the non-randomized approach. The covariates for adjustment were carefully chosen on the basis of prior knowledge of their relevance to the outcome. The effect of the OH subgroup, age, LDL-C, and the assessment of the frequency of hospital admission on the primary endpoint as continuous variables was determined. This was accomplished by illustrating limited cubic spline curves from the adjusted proportional hazards model. The study conducted sensitivity analyses using a competing risks model to evaluate the competing risks of other deaths with cardiovascular death. The Nelson–Aalen cumulative risk curves were utilized to illustrate cumulative incidence function (CIF), while Gray's test was employed to examine variability between groups. In addition, the association between OH and cardiovascular mortality was analyzed with the multifactor competing risk regression model, adjusting for the influence of competing risk events. Competing events in this study were defined as (1) individuals who did not experience cardiovascular death, (2) individuals who experienced cardiovascular death, and (3) individuals who died from other causes.

Statistical significance was defined as a two-sided *P*-value ≤ 0.05. The Statistical Package for the Social Sciences (SPSS) Statistics version 26 (IBM Corp., Armonk, NY, USA) and R software version 4.2.1 (R Foundation for Statistical Computing, Vienna, Austria) were adopted to make all analyses.

## 3 Results

### 3.1 Demographic data and baseline characteristics

For the 924 patients with stable CAD meeting the inclusion–exclusion criteria, the baseline features are provided in Table 1. Of these, 869 (94.05%) were men and 55 (5.95%) were women. There were 263 cases (28.46%) in the OH group and 661 cases (71.54%) in the non-OH group in the baseline population.

**Table 1:** Characteristics of patients with and without orthostatic hypotension.

Characteristic	Total (*n* = 924)	Subject without OH (*n* = 661)	Subject with OH (*n* = 263)	*P*-value
Anagraphics and history				
Male, *n* (%)	869 (94.0)	615 (93.0)	254 (96.6)	0.058
Age (years)	82.00 [80.00–85.00]	82.00 [80.00–84.00]	82.00 [80.00–85.00]	0.013
BMI (kg/m^2^)	24.82 [22.95–26.81]	24.77 [22.86–26.81]	25.00 [23.09–26.78]	0.324
Height (m)	168.00 [163.00–172.00]	167.00 [163.00–172.00]	168.00 [164.00–172.00]	0.202
Weight (kg)	70.00 [63.00–75.00]	70.00 [63.00–75.00]	70.00 [64.00–76.00]	0.109
WHR	0.92 [0.89–0.95]	0.91 [0.88–0.95]	0.92 [0.89–0.95]	0.130
Lifestyle				
Smoking, *n* (%)	157 (17.0)	105 (15.9)	52 (19.8)	0.186
Physical activity, *n* (%)				0.059
Some	132 (14.3)	87 (13.2)	45 (17.1)	
Fairly	721 (78.0)	516 (78.1)	205 (77.9)	
Quite	71 (7.7)	58 (8.8)	13 (4.9)	
Alcohol, *n* (%)	155 (16.8)	101 (15.3)	54 (20.5)	0.067
No-invasion resting hemodynamic				
HR (bpm)	70.00 [64.00–76.00]	70.00 [65.00–77.00]	68.00 [64.00–75.00]	0.066
SBP (mmHg)	130.00 [120.00–140.00]	130.00 [120.00–140.00]	130.00 [120.00–140.00]	0.246
DBP (mmHg)	70.00 [65.00–80.00]	70.00 [65.00–80.00]	70.00 [64.00–80.00]	0.417
PP (mmHg)	60.00 [50.00–70.00]	60.00 [50.00–68.00]	60.00 [50.00–70.00]	0.099
Laboratory data				
FBG (mmol/L)	5.40 [4.90–6.00]	5.40 [4.90–6.00]	5.40 [5.00–6.10]	0.353
Creatinine (μmol/L)	85.00 [74.00–101.25]	85.00 [73.00–100.00]	86.00 [76.50–106.00]	0.013
BUN (mmol/L)	6.50 [5.40–7.80]	6.40 [5.30–7.80]	6.60 [5.50–7.75]	0.161
UA (μmol/L)	380.50 [321.00–439.00]	379.00 [321.00–439.00]	386.00 [323.50–438.50]	0.535
TCHO (mmol/L)	4.62 (0.98)	4.61 (0.98)	4.65 (0.98)	0.540
HDL-C (mmol/L)	1.10 [0.94–1.29]	1.10 [0.93–1.30]	1.12 [0.94–1.28]	0.733
LDL-C (mmol/L)	2.85 [2.25–3.45]	2.82 [2.25–3.45]	2.94 [2.21–3.48]	0.659
TG (mmol/L)	1.36 [1.03–1.90]	1.38 [1.03–1.93]	1.29 [1.00–1.82]	0.495
eGFR (ml/min/1.73 m^2^)	79.79 [64.51–95.04]	81.07 [65.48–96.62]	78.75 [62.08–92.14]	0.026
Comorbidities				
Hypertension, *n* (%)	693 (75.0)	477 (72.2)	216 (82.1)	0.002
DM, *n* (%)	378 (40.9)	258 (39.0)	120 (45.6)	0.077
HF, *n* (%)	130 (14.1)	80 (12.1)	50 (19.0)	0.009
MI, *n* (%)	166 (18.0)	100 (15.1)	66 (25.1)	0.001
Stroke, *n* (%)	94 (10.2)	54 (8.2)	40 (15.2)	0.002
Parkinsonism, *n* (%)	237 (25.6)	154 (23.3)	83 (31.6)	0.012
Medications				
Anti-hypertensive drug, *n* (%)	597 (64.6)	404 (61.1)	193 (73.4)	0.001
Hypoglycemic drugs, *n* (%)	240 (26.0)	160 (24.2)	80 (30.4)	0.063

Data are presented as median [interquartile range], mean (standard deviation), or number of patients (%).

Differences in gender, BMI, WHR, smoking, physical activity, alcohol consumption, SBP, DBP, PP, FBG, BUN, UA, TCHO, HDL-C, LDL-C, TG, DM, and history of hypoglycemic medication usage were not statistically significant between the OH group and non-OH group (*P* > 0.05). Although both groups displayed the median age of 82 years, the OH group showed overall larger age than the non-OH group (*P* = 0.013). In addition, statistically important diversities were found in sitting HR, creatinine, eGFR, HF, MI, stroke, Parkinsonism, history of hypertension, and history of anti-hypertensive medication usage between the OH and non-OH groups (*P* < 0.05).

### 3.2 Disease progression and survival rates

This study had an observation deadline of 1 June 2023. The dropout rate was 2.27% (21/924) during the 159 months of follow-up, much less than 10%. The OH group showed the average follow-up duration of 116 months (IQR: 70–159), while it was 145 months (IQR: 79–159) for the non-OH group. During this period, the 1-, 5-, and 13-year readmission rates of the OH group for cardiovascular events, respectively, were 29.53%, 48.43%, and 65.35%, which were higher than that of the non-OH group (19.72%, 37.90%, and 51.62%).

**Figure 2:**
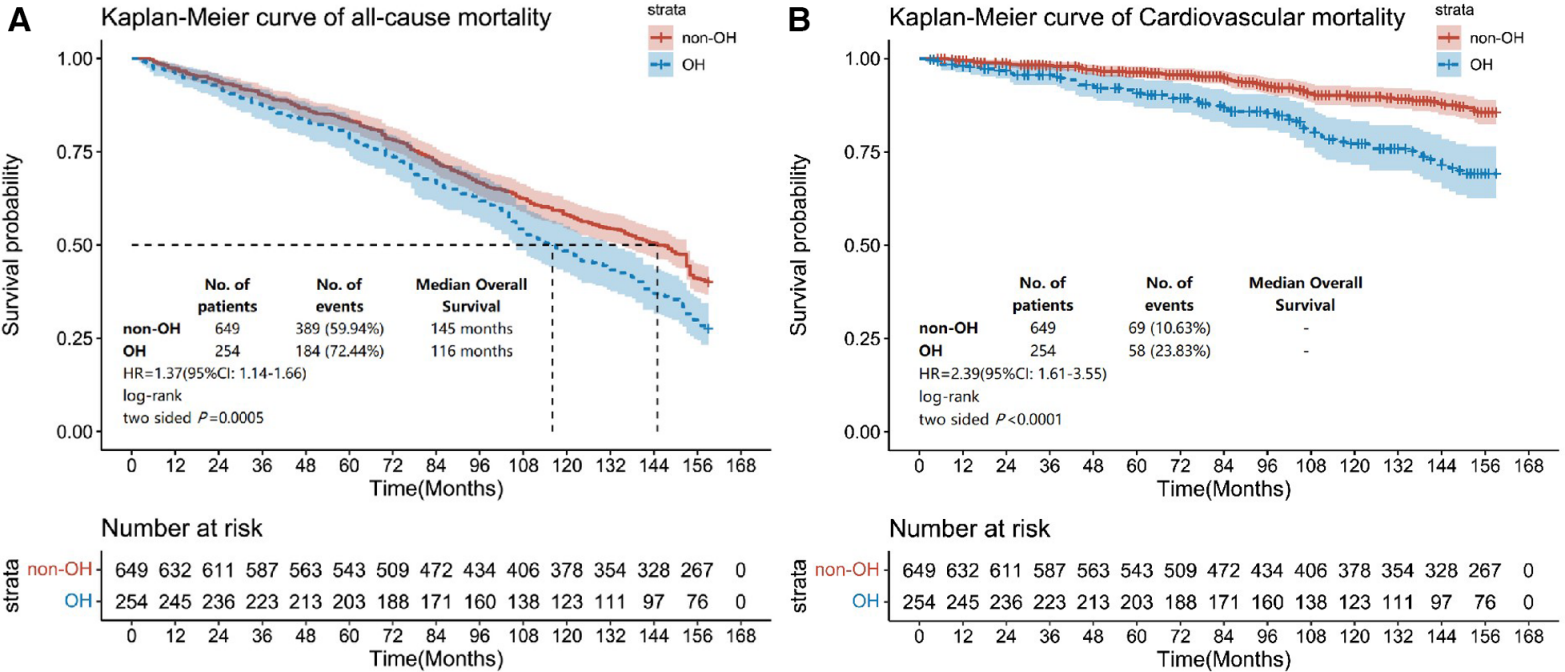
Kaplan–Meier survival curves for all-cause mortality and cardiovascular disease mortality based on subgroups with and without OH. The endpoint outcome event in (**A**) is all-cause mortality, and the endpoint outcome event in (**B**) is cardiovascular disease mortality.

The results indicated that the non-OH group exhibited an all-cause mortality rate of 59.94%, whereas the OH group showed a notably higher rate of 72.44%. It was found that the OH group showed 1.37 times greater risk of all-cause mortality than the non-OH group (HR = 1.37, 95% CI: 1.14–1.66) (Figure 2A). Furthermore, the non-OH group had a cardiovascular disease mortality rate of 10.63%, whereas the OH group had a markedly higher rate of 23.83%. The OH group exhibited a cardiovascular disease risk that was 2.39 times higher than that observed for the non-OH group (HR = 2.39, 95% CI: 1.61–3.55) (Figure 2B).

### 3.3 OH and all-cause mortality and CVD mortality

To investigate whether OH serves as a risk element for increasing all-cause mortality and cardiovascular death among patients with stable CAD, univariate Cox regression analyses were conducted (Table 2). Sex, age, BMI, WHR, smoking, alcohol consumption, exercise intensity, SBP, PP, FBG, creatinine, TCHO, HDL-C, LDL-C, eGFR, hypertension, DM, HF, MI, stroke, drug history for anti-hypertension, OH, OH_0_, and OH_2_ were included as variables. The results showed that OH, along with OH_0_ and OH_2_, hypertension, diabetes mellitus, as well as previous cardiovascular events, were risk factors for all-cause mortality (*P* < 0.05). To address the time-varying effects of BMI, we categorized BMI into two groups: overweight (BMI ≥ 25) and not overweight (BMI < 25). This transformation allowed us to satisfy the PH assumption for the variable and the overall model. We constructed three Cox regression models that were adjusted for sociodemographic characteristics, clinical indicators, and laboratory indicators. The stratified Cox proportional risk regression analyses revealed that only overweight hypertension had a statistically significant impact on all-cause mortality among individuals with a BMI greater than 25 in Models 1 and 2, with risk proportions of 1.40 (95% CI: 1.10–1.80) and 1.30 (95% CI: 1.00–1.70). However, in the adjusted final model (Model 3), OH, OH_0_, and OH_2_ did not show statistical significance (*P* > 0.05) (Table 3, Supplementary Tables S1–S3).

**Table 2:** Univariate cox regression analyses for all-cause mortality and cardiovascular disease mortality.

Variable	All-cause mortality		Cardiovascular disease mortality	
	HR (95% CI)	*P*-value	HR (95% CI)	*P*-value
Anagraphics and history				
Male, *n* (%)	1.81 (1.17–2.80)	0.008	2.06 (0.76–5.57)	0.155
Age (years)	1.13 (1.10–1.15)	<0.001	1.15 (1.10–1.20)	<0.001
BMI (Kg/m^2^)	0.92 (0.84–1.00)	0.044	0.96 (0.80–1.15)	0.667
WHR	1.15 (1.06–1.25)	0.001	1.14 (0.96–1.36)	0.132
Lifestyle				
Smoking, *n* (%)	1.72 (1.41–2.10)	<0.001	1.70 (1.11–2.58)	0.014
Physical activity, *n* (%)	0.62 (0.52–0.74)	<0.001	0.77 (0.53–1.13)	0.181
Some	1		1	
Fairly	0.80 (0.66–0.97)	0.020	0.98 (0.64–1.51)	0.938
Quite	0.64 (0.45–0.91)	0.012	0.70 (0.34–1.42)	0.320
Alcohol, *n* (%)	1.24 (1.00–1.53)	0.046	1.69 (1.12–2.55)	0.012
No-invasion resting hemodynamic				
HR (bpm)	1.07 (0.99–1.16)	0.091	0.97 (0.81–1.15)	0.703
SBP (mmHg)	1.10 (1.01–1.19)	0.032	1.35 (1.15–1.59)	<0.001
DBP (mmHg)	0.99 (0.91–1.08)	0.820	1.00 (0.83–1.19)	0.960
PP (mmHg)	1.11 (1.02–1.20)	0.015	1.39 (1.18–1.63)	<0.001
Laboratory data				
FBG (mmol/L)	1.13 (1.05–1.22)	0.001	1.16 (0.99–1.35)	0.061
Creatinine (μmol/L)	1.17 (1.10–1.24)	<0.001	1.18 (1.04–1.33)	0.008
BUN (mmol/L)	1.03 (0.98–1.09)	0.216	1.06 (0.97–1.15)	0.208
UA (μmol/L)	1.07 (0.98–1.16)	0.138	1.16 (0.98–1.38)	0.092
TCHO (mmol/L)	0.91 (0.84–0.99)	0.036	0.81 (0.67–0.97)	0.019
HDL-C (mmol/L)	0.91 (0.83–0.99)	0.035	0.75 (0.61–0.91)	0.004
LDL-C (mmol/L)	0.96 (0.89–1.04)	0.36	1.27 (1.07–1.51)	0.006
TG (mmol/L)	0.99 (0.91–1.08)	0.865	0.85 (0.69–1.06)	0.150
eGFR (ml/min/1.73 m^2^)	0.80 (0.70–0.91)	0.001	0.68 (0.52–0.91)	0.009
Comorbidities				
Hypertension, *n* (%)	1.64 (1.33–2.02)	<0.001	1.93 (1.20–3.12)	0.007
DM, *n* (%)	1.26 (1.07–1.49)	0.006	1.66 (1.17–2.35)	0.004
HF, *n* (%)	2.33 (1.90–2.85)	<0.001	5.30 (3.70–7.61)	<0.001
MI, *n* (%)	1.47 (1.21–1.80)	<0.001	4.11 (2.89–5.85)	<0.001
Stroke, *n* (%)	1.67 (1.40–1.99)	<0.001	1.42 (0.97–2.09)	0.072
Parkinsonism, *n* (%)	1.13 (0.88–1.46)	0.343	0.96 (0.54–1.70)	0.887
Medications				
Anti-hypertensive drug, *n* (%)	0.94 (0.80–1.12)	0.498	1.21 (0.83–1.76)	0.327
Hypoglycemic drugs, *n* (%)	1.42 (1.19–1.70)	<0.001	1.63 (1.13–2.35)	0.009
OH	1.37 (1.15–1.64)	<0.001	2.39 (1.69–3.40)	<0.001
OH_0_	1.26 (1.04–1.53)	0.016	1.78 (1.23–2.59)	0.002
OH_2_	1.23 (1.03–1.47)	0.019	1.96 (1.38–2.79)	<0.001

**Table 3:** Relative risk of all-cause mortality in patients with stable coronary artery disease from orthostatic hypotension.

	Model 1		Model 2		Model 3	
	HR (95% CI)	*P*-value	HR (95% CI)	*P*-value	HR (95% CI)	*P*-value
BMI < 25						
OH	1.10 (0.89–1.50)	0.310	1.00 (0.81–1.30)	0.750	1.10 (0.82–1.40)	0.660
OH_0_	1.10 (0.88–1.50)	0.320	1.00 (0.78–1.40)	0.820	1.10 (0.80–1.40)	0.700
OH_2_	0.98 (0.76–1.20)	0.840	0.93 (0.72–1.20)	0.580	0.93 (0.72–1.20)	0.560
BMI ≥ 25						
OH	1.40 (1.10–1.80)	0.008	1.30 (1.00–1.70)	0.047	1.30 (0.98–1.70)	0.069
OH_0_	1.20 (0.93–1.60)	0.150	1.30 (0.95–1.70)	0.110	1.20 (0.91–1.60)	0.180
OH_2_	1.20 (0.94–1.60)	0.150	1.20 (0.89–1.50)	0.270	1.10 (0.87–1.50)	0.360

Model 1: adjusting for sex, age, body mass index, waist-to-hip ratio, smoking, physical activity, and alcohol. Model 2: adjusting for sex, age, body mass index, waist-to-hip ratio, smoking, physical activity, alcohol, systolic blood pressure, pulse pressure, hypertension, diabetes mellitus, heart failure, myocardial infarction, stroke, and hypoglycemic drugs. Model 3: adjusting for sex, age, body mass index, waist-to-hip ratio, smoking, physical activity, alcohol, systolic blood pressure, pulse pressure, hypertension, diabetes mellitus, heart failure, myocardial infarction, stroke, hypoglycemic drugs, fasting blood glucose, creatinine, total cholesterol, high-density lipoprotein cholesterol, low-density lipoprotein cholesterol, and estimated glomerular filtration rate.

To explore the independent association between OH and cardiovascular mortality in patients diagnosed with stable CAD, one-way Cox regression (Table 2) and multifactorial Cox proportional hazards models (Table 4, Supplementary Tables S4–S6) were employed. Our results demonstrated that OH was related to a growing risk of cardiovascular disease death in all three adjusted models (*P* < 0.05). Furthermore, even after adjusting confounding elements, the final model revealed a 1.80-fold grown risk of cardiovascular disease death in study subjects with OH (95% CI: 1.20–2.60). In addition, it was found that both OH_0_ and OH_2_ were significantly related to the risk of cardiovascular disease death (*P* < 0.05), providing further evidence that OH might be an isolated risk element for cardiovascular disease death.

**Table 4:** Relative risk of CVD mortality in patients with stable CAD from orthostatic hypotension.

	Model 1		Model 2		Model 3	
	HR (95%CI)	*P*-value	HR (95% CI)	*P*-value	HR (95% CI)	*P*-value
OH	2.20 (1.50–3.10)	<0.001	1.80 (1.30–2.60)	0.001	1.80 (1.20–2.60)	0.001
OH_0_	1.70 (1.20–2.40)	0.005	1.50 (1.00–2.10)	0.028	1.50 (1.00–2.10)	0.037
OH_2_	1.70 (1.20–2.50)	0.006	1.60 (1.10–2.40)	0.009	1.50 (1.00–2.20)	0.034

Model 1: adjusting for age, smoking, and alcohol consumption. Model 2: adjusting for age, smoking, alcohol consumption, systolic blood pressure, pulse pressure, hypertension, diabetes mellitus, heart failure, myocardial infarction, and hypoglycemic drugs. Model 3: adjusting for age, smoking, alcohol consumption, systolic blood pressure, pulse pressure, hypertension, diabetes mellitus, heart failure, myocardial infarction, hypoglycemic drugs, creatinine, total cholesterol, high-density lipoprotein cholesterol, low-density lipoprotein cholesterol, and estimated glomerular filtration rate.

**Figure 3:**
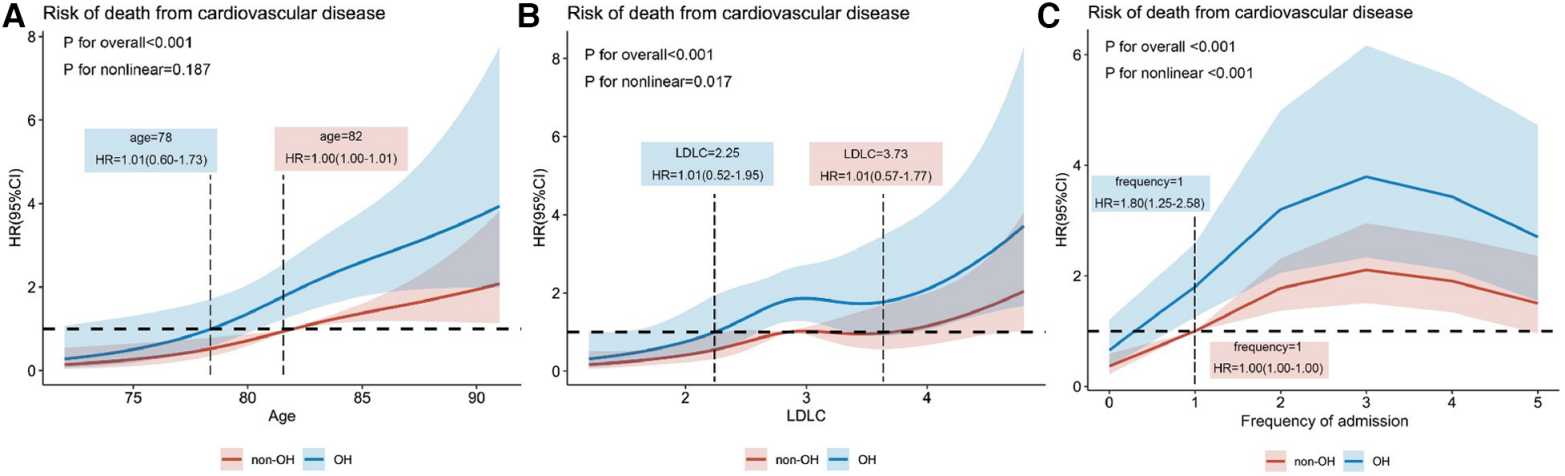
Relationships of age (**A**), LDL-C (**B**), and frequency of admission (**C**) to the hazard ratio for death from CVDs explained by adjusted restricted cubic spline plots.

The 159-month risk of cardiovascular death was influenced by age, LDL concentration, and frequency of readmission. The adjusted Cox regression model, depicted by restricted cubic spline curves, showed a significant overall model with a *P* < 0.001. The non-linearity test showed *P*-value of 0.187, 0.017, and <0.001 for the three graphs, respectively. The reference value (HR = 1) was the threshold for the risk of cardiovascular death. As shown in Figure 3A, cardiovascular death risk increased with age and occurs earlier in the OH group in comparison with the non-OH group (ages: 78 and 82 years). In Figure 3B, it is seen that the risk of cardiovascular death rises with increasing LDL-C concentration, particularly when LDL-C is greater than 2.25 and 3.73 mmol/L in the OH and non-OH groups, respectively. In addition, as displayed in Figure 3C, the frequency of hospital admissions for cardiovascular diseases was an important factor in cardiovascular death and the risk of readmission was at least 1.8 times higher in the OH group than in the non-OH group, with the former having a significantly higher frequency of admissions than the latter.

### 3.4 Sensitivity analyses

**Figure 4:**
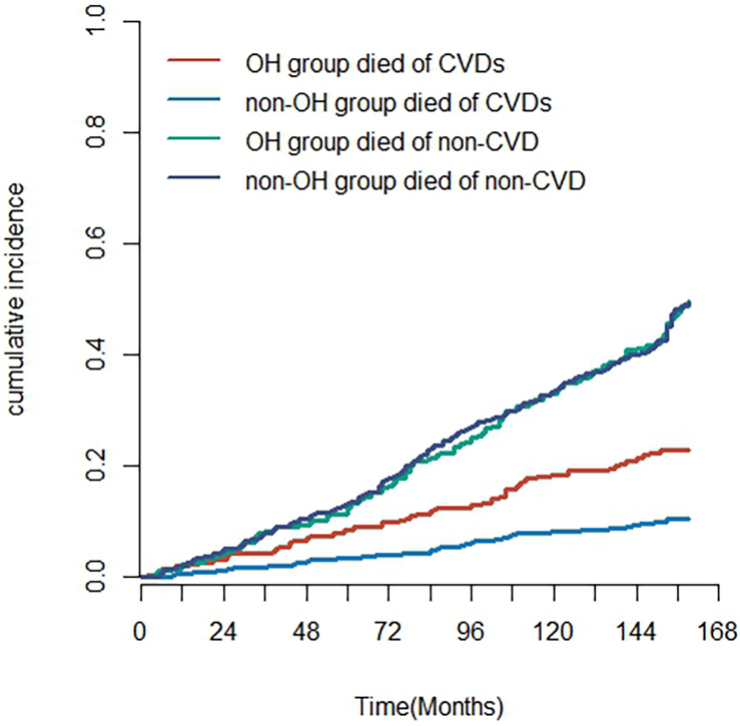
The cumulative incidence of deaths in the OH and non-OH groups from CVD and competing risk events (non-CVD).

The Nelson–Aalen cumulative risk curves indicate the cumulative incidence of deaths in the OH and non-OH groups (Figure 4). Gray's test revealed a significantly higher cumulative incidence of cardiovascular disease-related mortality in the postural hypotension group in comparison with the group without postural hypotension (*Z* = 22.811, *P* < 0.001). However, no statistically important diversity was observed in the cumulative incidence of non-cardiovascular disease-related mortality between the two groups (*Z* = 0.001, *P* = 0.983). In cause-specific risk regression, OH was identified as a risk element for cardiovascular disease death [subdistribution risk ratio (sHR) = 1.74 (1.22–2.49), *P* = 0.002] but not for non-cardiovascular disease death (considered as a competing risk event) [sHR = 1.04 (0.84–1.27), *P* = 0.738] (Supplementary Table S7).

After adjusting for the potential influencing factors of OH, age, alcohol consumption, SBP, PP, DM, HF, MI, TCHO, HDL-C, LDL-C, and use of anti-hyperglycemic drugs, the results of the multifactorial CIF regression model function indicated a greater incidence of cardiovascular death in the OH group in comparison with the non-OH group [sHR = 1.91 (1.30–2.70), *P* < 0.001], which aligns with the findings from the multifactorial Cox regression analysis. Notably, age emerged as an important risk factor for cardiovascular disease death even after accounting for competing risk events [sHR = 1.10 (1.00–1.20), *P* < 0.001] (Supplementary Table S8).

## 4 Discussion

This study aimed at assessing the correlation between all-cause mortality and cardiovascular death among patients with stable CAD and OH. Our study shown that compared with the non-OH group, patients in the OH group had higher risk of all-cause mortality when confounding factors was not adjusted. But after adjusting for confounding factors, the relationship between these two groups was not significant, meaning that OH could not be considered as a predictor for all-cause mortality in the elderly. Identical conclusions were reported in a meta-analysis research involving 10 cohort research studies (HR: 1.26, 95% CI: 0.99–1.62) (11), and many prospective studies also found no direct association between a single OH and in-hospital all-cause mortality (18, 19). But some of the studies are not in a line with us (20–23). There are two possible reasons for the inconsistency: one is that this study was based on a group of elderly and vulnerable people who live permanently in nursing homes, the other is that the BMI stratification was implemented to fit the PH presumptive test, leading to a smaller sample size, potentially contributing to the lack of favorable outcomes.

In addition, our study revealed a particularly significant effect of OH on cardiovascular death, and several cohort studies with shorter follow-up periods have also presented results consistent with ours (24–27). However, the study of Casiglia et al. (28) revealed that OH may not serve as a reliable prognosticator of cardiovascular events (HR: 1.33, 95% CI: 0.78–2.20). This could be due to the variability in blood pressure among individuals, leading to a less clear link between blood pressure variability and the risk of cardiovascular events in the general population compared with patients with stable CAD.

Several mechanisms may be based on the relationship between the prevalence of OH and a greater risk of cardiovascular events. First, OH disrupts hemodynamic stability and diminishes the ability of buffer blood pressure changes and counteract compensatory adjustments in cardiovascular remodeling, leading to slower blood flow (29–31), which can predispose to thrombus formation and increase the risk of cardiovascular events such as myocardial infarction (32, 33). Second, OH is a major sign of autonomic dysfunction (34). In instances where blood fails to efficiently reach the upper body upon standing, compromised tissue perfusion may occur, potentially triggering cardiovascular events like myocardial ischemia (27). In addition, proteomic studies have identified MMP-7, MB, TM, and TIM-1 as biomarkers associated with atherosclerotic thrombosis and inflammation associated with OH (35). This highlights the importance of identifying and effectively managing OH to mitigate the risk of cardiovascular events in the affected population.

The characteristics of the susceptible population are discussed next. As previously reported, the risk of cardiovascular death tends to rise with age (36). Our study further indicates that this risk is particularly pronounced among older adults with OH. Elevated LDL-C, a recognized cardiovascular event risk factor, presents a noteworthy variation in the OH population, whose risk thresholds are lower than the traditional clinical one of 3.12 mmol/L. This emphasizes the imperative for effective lipid control specifically tailored to individuals with OH. In a global epidemiological study of dyslipidemia, elevated plasma LDL-C levels were shown to be a major causative factor for ischemic heart disease in both developed and developing countries and ranked eighth among the major risk factors for death in 2019 (37). Moreover, insights from the Utrecht Cardiovascular Cohort–SMART cohort study revealed an escalating incidence of cardiovascular events corresponding to the number of symptomatic arterial disease sites (such as CAD, CVD, and peripheral arterial disease), consequently elevating the risk of death (38). Our subgroup analyses corroborated these findings, highlighting the frequency of cardiovascular event admissions as a distinct risk factor for cardiovascular death. Notably, individuals in the OH group faced a risk of death approximately twice that of those in the non-OH group.

Furthermore, OH is often associated with other comorbidities such as diabetes, hypertension, heart failure, and myocardial infarction, which are all risk factors for cardiovascular events (39). In the treatment and management of elderly patients with multiple underlying diseases and comorbidities, especially those with comorbid hypertension (34), the risk of supine hypertension that may be associated with excessive OH therapies should be considered (40, 41), and therefore, gathering insights into the pathomechanisms of neurogenic, non-neurogenic, and mixed OH is the next step in the research program. In addition, long-term management remains a challenging issue for most elderly patients (42), during which patient education is essential to alleviate upright intolerance, but it is often overlooked or underestimated. Patients and families should have a basic knowledge of upright physiology and an understanding of non-pharmacological treatments to develop effective strategies against BP reduction (43, 44). In summary, a comprehensive treatment and management strategy is key to the care of patients with stable CAD and comorbid OH, and the assessment of the severity and frequency of patient symptoms is a prerequisite.

## 5 Strengths and limitations

First, even though it is limited to a single-center study with limited population coverage, our study population has similar work experiences, lifestyle habits, and environmental factors, which reduces the potential influence of external factors on the results of the study, and also reduces the likelihood of extrapolating the findings of the study to the general population. In addition, the dropout rate is low because they visit our hospital for yearly physicals, and our hospital serves as their designated healthcare facility. This enables us to collect precise and comprehensive clinical and survival data related to them.

Second, no long-term study has investigated the effects of OH on a population with stable coronary heart disease, likely due to a short follow-up period that is insufficient for observing cardiovascular death outcomes adequately. But our study was conducted over 13 years of follow-up, including analyses with both multivariate Cox proportional risk assumptions and competing risk models. For all we know, this study is the longest follow-up research currently made in China within the pertinent research domains. Notably, it marks the pioneering effort to examine OH and the long-term prognosis of CAD in a population of retired military personnel. However, the study's findings can only partially represent men, thus limiting the generalizability of the conclusions. This is primarily because there are more men than women in the military. Hence, additional research on women is required.

Third, our study did not obtain the result that a history of anti-hypertensive medication use had a positive effect on all-cause mortality and cardiovascular deaths in patients, which does not support the current practice guidelines for blood pressure medication (45), the main reason for which could be the fact that some patients with hypertension do not take anti-hypertensive medication as prescribed, thus leading to an underestimation of the positive effect of taking anti-hypertensive medication on the long-term prognosis in the univariate survival analyses. However, it is unfortunate that patients’ medication use was not recorded during follow-up.

Lastly, we acknowledge that after carefully controlling for variables, we were unable to find any correlation between OH and all-cause mortality. This is likely since we used stratified variables to satisfy the PH hypothesis test, which reduced the sample size. Therefore, additional data must be gathered to validate our conclusions and further support them.

## 6 Conclusion

In this study, we shared findings from a cohort study tracking patients with stable CAD compounded by OH over a long-term follow-up. Our conclusion highlights a notably elevated risk of cardiovascular death among those with stable CAD and OH compared with those solely diagnosed with stable CAD. However, it's important to note that this correlation did not emerge in multivariate survival analyses where all-cause mortality served as the primary endpoint.

## Data Availability

The data analyzed in this study is subject to the following licenses/restrictions: The population for this study is a profile of retired veteran cadres from the Chinese military, so these data were not allowed to be issued by the Ethics Committee of the General Hospital of Southern Theater Command. Requests to access these datasets should be directed to Jiaman Hu, hujiaman@163.com.
