# Supplementary material for: Effect of orthostatic hypotension on long-term prognosis of elderly patients with stable coronary artery disease: a retrospective cohort study
Source: Front Cardiovasc Med. 2024 Apr 12;11:1342379. doi: 10.3389/fcvm.2024.1342379 (PMC11048043; doi:10.3389/fcvm.2024.1342379)
Supplement: Supplementary file 1 [file Table1.docx]

Supplementary Material

# Supplementary Data

**Supplementary Table 1**  Relative Risk of All-cause Mortality in Patients with Stable Coronary Artery Disease by OH

**Supplementary Table 2** Relative Risk of All-cause Mortality in Patients with Stable Coronary Artery Disease by OH 0

**Supplementary Table 3** Relative Risk of All-cause Mortality in Patients with Stable Coronary Artery Disease by OH 2

**Supplementary Table 4** Relative Risk of Cardiovascular disease mortality in Patients with Stable Coronary Artery Disease by OH

**Supplementary Table 5** Relative Risk of Cardiovascular disease mortality in Patients with Stable Coronary Artery Disease by OH 0

**Supplementary Table 6** Relative Risk of Cardiovascular disease mortality in Patients with Stable Coronary Artery Disease by OH 2

**Supplementary Table 7** Cause-specific risk regression modelling results

**Supplementary Table 8** Univariate and multivariate competition risk modelling for Cardiovascular Disease Mortality

| **Abbreviations** | |
| --- | --- |
| OH | orthostatic hypotension |
| BMI | Body mass index |
| WHR | waist-to-hip ratio |
| SBP | systolic blood pressure |
| PP | pulse pressure |
| DM | diabetes mellitus |
| HF | heart failure |
| MI | myocardial infarction |
| FBG | fasting blood glucose |
| TCHO | total cholesterol |
| HDLC | high density lipoprotein cholesterol |
| LDLC | low density lipoprotein cholesterol |
| eGFR | estimating glomerular filtration rate |

# Supplementary Tables

**
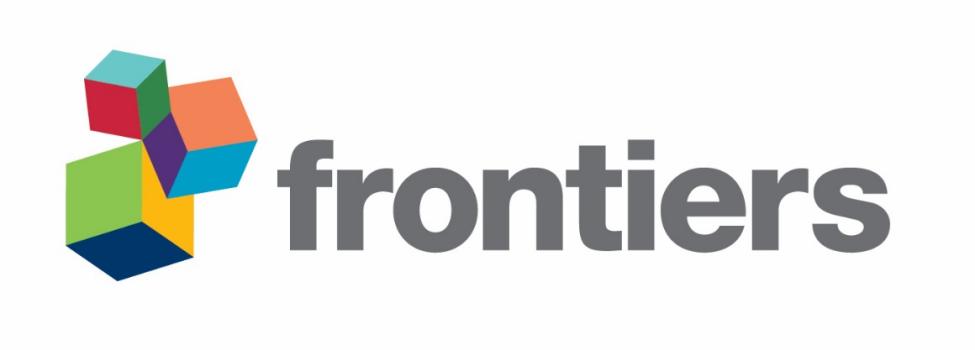
**

| **Supplementary Table 1**  Relative Risk of All-cause Mortality in Patients with Stable Coronary Artery Disease by OH | | | | | | | | | | | | | |
| --- | --- | --- | --- | --- | --- | --- | --- | --- | --- | --- | --- | --- | --- |
| variable | Model 1 | | | | Model 2 | | | | Model 3 | | | | |
|  | HR(95%CI) | *P*-value | HR(95%CI) | *P*-value | HR(95%CI) | *P*-value | HR(95%CI) | *P*-value | HR(95%CI) | *P*-value | HR(95%CI) | *P*-value |  |
|  | **BMI<25** |  | **BMI≥25** |  | **BMI<25** |  | **BMI≥25** |  | **BMI<25** |  | **BMI≥25** |  |  |
| OH | 1.10 (0.89, 1.50) | 0.310 | 1.40 (1.10, 1.80) | 0.008 | 1.00 (0.81, 1.30) | 0.750 | 1.30 (1.00, 1.70) | 0.047 | 1.10 (0.82, 1.40) | 0.660 | 1.30 (0.98, 1.70) | 0.069 |  |
| Sex | 1.80 (1.10, 3.00) | 0.019 | 2.00 (0.73, 5.40) | 0.180 | 1.60 (0.99, 2.70) | 0.053 | 2.10 (0.76, 5.70) | 0.160 | 1.70 (1.00, 2.80) | 0.047 | 1.70 (0.63, 4.80) | 0.280 |  |
| Age | 1.10 (1.10, 1.10) | <0.001 | 1.20 (1.10, 1.20) | <0.001 | 1.10 (1.10, 1.10) | <0.001 | 1.10 (1.10, 1.20) | <0.001 | 1.10 (1.10, 1.10) | <0.001 | 1.20 (1.10, 1.20) | <0.001 |  |
| BMI (per 1 SD) | 0.78 (0.65, 0.95) | 0.015 | 1.10 (0.89, 1.30) | 0.490 | 0.77 (0.64, 0.94) | 0.011 | 1.00 (0.84, 1.20) | 0.900 | 0.78 (0.64, 0.95) | 0.016 | 1.00 (0.83, 1.20) | 0.970 |  |
| WHR (per 1 SD) | 1.10 (0.97, 1.30) | 0.140 | 1.20 (1.10, 1.40) | 0.001 | 1.10 (0.94, 1.20) | 0.320 | 1.30 (1.10, 1.40) | 0.000 | 1.00 (0.91, 1.20) | 0.510 | 1.30 (1.10, 1.40) | <0.001 |  |
| Smoking | 1.20 (0.89, 1.60) | 0.240 | 1.40 (1.00, 2.00) | 0.037 | 1.20 (0.87, 1.60) | 0.320 | 1.30 (0.90, 1.80) | 0.180 | 1.20 (0.87, 1.60) | 0.280 | 1.30 (0.90, 1.80) | 0.170 |  |
| Physical activity | 0.56 (0.44, 0.71) | <0.001 | 0.72 (0.54, 0.98) | 0.034 | 0.58 (0.44, 0.75) | <0.001 | 0.71 (0.52, 0.97) | 0.031 | 0.58 (0.44, 0.75) | <0.001 | 0.73 (0.53, 1.00) | 0.052 |  |
| some | - | - | - | - | - | - | - | - | - | - | - | - |  |
| fairly | 0.51 (0.38, 0.67) | <0.001 | 0.60 (0.43, 0.85) | 0.004 | 0.52 (0.39, 0.71) | <0.001 | 0.58 (0.40, 0.84) | 0.004 | 0.52 (0.39, 0.71) | <0.001 | 0.58 (0.40, 0.85) | 0.005 |  |
| quite | 0.37 (0.22, 0.64) | <0.001 | 0.67 (0.37, 1.20) | 0.180 | 0.39 (0.22, 0.68) | 0.001 | 0.61 (0.34, 1.10) | 0.110 | 0.39 (0.22, 0.69) | 0.001 | 0.69 (0.37, 1.30) | 0.230 |  |
| Alcohol | 1.20 (0.88, 1.60) | 0.270 | 1.30 (0.94, 1.80) | 0.110 | 1.20 (0.91, 1.70) | 0.190 | 1.40 (1.00, 1.90) | 0.033 | 1.20 (0.92, 1.70) | 0.160 | 1.40 (0.99, 1.90) | 0.057 |  |
| SBP (per 1 SD) |  |  |  |  | 1.20 (0.99, 1.40) | 0.073 | 1.10 (0.87, 1.30) | 0.570 | 1.20 (0.97, 1.40) | 0.100 | 1.00 (0.85, 1.20) | 0.830 |  |
| PP (per 1 SD) |  |  |  |  | 0.99 (0.83, 1.20) | 0.910 | 0.90 (0.75, 1.10) | 0.280 | 0.99 (0.83, 1.20) | 0.940 | 0.91 (0.76, 1.10) | 0.320 |  |
| Hypertention |  |  |  |  | 0.85 (0.65, 1.10) | 0.260 | 1.10 (0.81, 1.50) | 0.520 | 0.82 (0.62, 1.10) | 0.160 | 1.10 (0.78, 1.50) | 0.660 |  |
| DM |  |  |  |  | 1.10 (0.82, 1.60) | 0.430 | 1.00 (0.70, 1.50) | 0.880 | 1.10 (0.78, 1.60) | 0.550 | 1.00 (0.69, 1.50) | 0.920 |  |
| HF |  |  |  |  | 1.50 (1.10, 2.10) | 0.005 | 2.00 (1.50, 2.80) | <0.001 | 1.60 (1.10, 2.10) | 0.005 | 1.90 (1.30, 2.60) | <0.001 |  |
| MI |  |  |  |  | 1.10 (0.84, 1.50) | 0.450 | 1.70 (1.20, 2.30) | 0.001 | 1.10 (0.84, 1.50) | 0.440 | 1.60 (1.20, 2.20) | 0.002 |  |
| Stroke |  |  |  |  | 1.40 (1.10, 1.80) | 0.008 | 1.20 (0.87, 1.60) | 0.300 | 1.50 (1.10, 1.90) | 0.004 | 1.10 (0.83, 1.50) | 0.470 |  |
| Hypoglycaemic drugs |  |  |  |  | 1.40 (0.93, 2.00) | 0.110 | 1.20 (0.77, 1.70) | 0.480 | 1.30 (0.89, 1.90) | 0.180 | 1.10 (0.71, 1.60) | 0.730 |  |
| FBG (per 1 SD) |  |  |  |  |  |  |  |  | 1.10 (0.94, 1.20) | 0.330 | 1.00 (0.91, 1.20) | 0.620 |  |
| Creatinine (per 1 SD) |  |  |  |  |  |  |  |  | 1.10 (0.96, 1.20) | 0.240 | 1.80 (1.40, 2.30) | <0.001 |  |
| TCHO (per 1 SD) |  |  |  |  |  |  |  |  | 1.00 (0.85, 1.20) | 0.900 | 0.95 (0.75, 1.20) | 0.660 |  |
| HDLC (per 1 SD) |  |  |  |  |  |  |  |  | 0.96 (0.84, 1.10) | 0.540 | 0.97 (0.83, 1.10) | 0.680 |  |
| LDLC (per 1 SD) |  |  |  |  |  |  |  |  | 0.98 (0.82, 1.20) | 0.840 | 1.10 (0.88, 1.40) | 0.390 |  |
| eGFR (per 1 SD) |  |  |  |  |  |  |  |  | 0.95 (0.79, 1.10) | 0.570 | 1.40 (1.10, 1.90) | 0.020 |  |

Model 1: adjusting for sex, age, body mass index, waist-to-hip ratio, smoking, physical activity, alcohol

Model 2: adjusting for sex, age, body mass index, waist-to-hip ratio, smoking, physical activity, alcohol, systolic blood pressure, pulse pressure, hypertention, diabetes mellitus , heart failure, myocardial infarction, stroke, Hypoglycaemic drugs

Model 3: adjusting for sex, age, body mass index, waist-to-hip ratio, smoking, physical activity, alcohol, systolic blood pressure, pulse pressure, hypertention, diabetes mellitus , heart failure, myocardial infarction, stroke, Hypoglycaemic drugs, fasting blood glucose, creatinine, total cholesterol, high density lipoprotein cholesterol, low density lipoprotein cholesterol, estimating glomerular filtration rate.

| **Supplementary Table 2** Relative Risk of All-cause Mortality in Patients with Stable Coronary Artery Disease by OH 0 | | | | | | | | | | | | | |
| --- | --- | --- | --- | --- | --- | --- | --- | --- | --- | --- | --- | --- | --- |
| variable | Model 1 | | | | | Model 2 | | | | Model 3 | | | |
|  | HR(95%CI) | *P*-value | HR(95%CI) | *P*-value | HR(95%CI) | | *P*-value | HR(95%CI) | *P*-value | HR(95%CI) | *P*-value | HR(95%CI) | *P*-value |
|  | **BMI<25** |  | **BMI≥25** |  | **BMI<25** | |  | **BMI≥25** |  | **BMI<25** |  | **BMI≥25** |  |
| OH 0 | 1.10 (0.88, 1.50) | 0.320 | 1.20 (0.93, 1.60) | 0.150 | 1.00 (0.78, 1.40) | | 0.820 | 1.30 (0.95, 1.70) | 0.110 | 1.10 (0.80, 1.40) | 0.700 | 1.20 (0.91, 1.60) | 0.180 |
| Sex | 1.80 (1.10, 3.00) | 0.019 | 2.00 (0.75, 5.50) | 0.170 | 1.60 (0.99, 2.70) | | 0.054 | 2.10 (0.76, 5.70) | 0.150 | 1.70 (1.00, 2.80) | 0.047 | 1.70 (0.63, 4.80) | 0.280 |
| Age | 1.10 (1.10, 1.10) | <0.001 | 1.20 (1.10, 1.20) | <0.001 | 1.10 (1.10, 1.10) | | <0.001 | 1.10 (1.10, 1.20) | <0.001 | 1.10 (1.10, 1.10) | <0.001 | 1.20 (1.10, 1.20) | <0.001 |
| BMI (per 1 SD) | 0.79 (0.65, 0.96) | 0.016 | 1.10 (0.88, 1.30) | 0.560 | 0.77 (0.64, 0.94) | | 0.011 | 1.00 (0.83, 1.20) | 0.930 | 0.78 (0.64, 0.96) | 0.017 | 0.99 (0.82, 1.20) | 0.960 |
| WHR (per 1 SD) | 1.10 (0.97, 1.30) | 0.140 | 1.20 (1.10, 1.40) | 0.001 | 1.10 (0.93, 1.20) | | 0.330 | 1.30 (1.10, 1.40) | <0.001 | 1.00 (0.91, 1.20) | 0.530 | 1.30 (1.10, 1.50) | 0.000 |
| Smoking | 1.20 (0.89, 1.60) | 0.230 | 1.40 (1.00, 2.00) | 0.025 | 1.20 (0.87, 1.60) | | 0.320 | 1.30 (0.90, 1.80) | 0.180 | 1.20 (0.87, 1.60) | 0.280 | 1.30 (0.91, 1.80) | 0.150 |
| Physical activity | 0.56 (0.44, 0.71) | <0.001 | 0.71 (0.53, 0.96) | 0.026 | 0.58 (0.44, 0.75) | | <0.001 | 0.71 (0.52, 0.96) | 0.025 | 0.58 (0.44, 0.75) | <0.001 | 0.72 (0.53, 0.99) | 0.040 |
| some | - | - | - | - | - | | - | - | - | - | - | - | - |
| fairly | 0.50 (0.38, 0.67) | <0.001 | 0.60 (0.42, 0.85) | 0.004 | 0.52 (0.39, 0.71) | | <0.001 | 0.58 (0.40, 0.84) | 0.004 | 0.52 (0.39, 0.71) | <0.001 | 0.58 (0.40, 0.85) | 0.005 |
| quite | 0.37 (0.22, 0.64) | <0001 | 0.63 (0.35, 1.10) | 0.130 | 0.39 (0.22, 0.68) | | 0.001 | 0.61 (0.34, 1.10) | 0.110 | 0.39 (0.22, 0.69) | 0.001 | 0.65 (0.36, 1.20) | 0.170 |
| Alcohol | 1.20 (0.88, 1.60) | 0.260 | 1.30 (0.96, 1.80) | 0.091 | 1.20 (0.91, 1.70) | | 0.190 | 1.40 (1.00, 1.90) | 0.033 | 1.20 (0.92, 1.70) | 0.160 | 1.40 (1.00, 1.90) | 0.046 |
| SBP (per 1 SD) |  |  |  |  | 1.20 (0.99, 1.40) | | 0.073 | 1.10 (0.87, 1.30) | 0.570 | 1.20 (0.97, 1.40) | 0.110 | 1.00 (0.86, 1.30) | 0.710 |
| PP (per 1 SD) |  |  |  |  | 0.99 (0.83, 1.20) | | 0.910 | 0.90 (0.75, 1.10) | 0.280 | 0.99 (0.83, 1.20) | 0.950 | 0.90 (0.75, 1.10) | 0.280 |
| Hypertention |  |  |  |  | 0.85 (0.65, 1.10) | | 0.260 | 1.10 (0.81, 1.50) | 0.520 | 0.82 (0.62, 1.10) | 0.180 | 1.10 (0.79, 1.50) | 0.620 |
| DM |  |  |  |  | 1.10 (0.82, 1.60) | | 0.430 | 1.00 (0.70, 1.50) | 0.880 | 1.10 (0.78, 1.60) | 0.540 | 1.00 (0.71, 1.50) | 0.860 |
| HF |  |  |  |  | 1.50 (1.10, 2.10) | | 0.005 | 2.00 (1.50, 2.80) | <0.001 | 1.60 (1.10, 2.10) | 0.005 | 1.90 (1.40, 2.70) | <0.001 |
| MI |  |  |  |  | 1.10 (0.84, 1.50) | | 0.450 | 1.70 (1.20, 2.30) | 0.001 | 1.10 (0.84, 1.50) | 0.420 | 1.70 (1.20, 2.30) | 0.002 |
| Stroke |  |  |  |  | 1.40 (1.10, 1.80) | | 0.008 | 1.20 (0.87, 1.60) | 0.300 | 1.50 (1.10, 1.90) | 0.005 | 1.10 (0.84, 1.50) | 0.410 |
| Hypoglycaemic drugs |  |  |  |  | 1.40 (0.93, 2.00) | | 0.110 | 1.20 (0.77, 1.70) | 0.480 | 1.30 (0.89, 1.90) | 0.180 | 1.10 (0.71, 1.60) | 0.700 |
| FBG (per 1 SD) |  |  |  |  |  | |  |  |  | 1.10 (0.94, 1.20) | 0.340 | 1.00 (0.91, 1.20) | 0.710 |
| Creatinine (per 1 SD) |  |  |  |  |  | |  |  |  | 1.10 (0.96, 1.20) | 0.240 | 1.80 (1.40, 2.30) | <0.001 |
| TCHO (per 1 SD) |  |  |  |  |  | |  |  |  | 1.00 (0.85, 1.20) | 0.860 | 0.95 (0.76, 1.20) | 0.690 |
| HDLC (per 1 SD) |  |  |  |  |  | |  |  |  | 0.96 (0.84, 1.10) | 0.530 | 0.98 (0.84, 1.10) | 0.790 |
| LDLC (per 1 SD) |  |  |  |  |  | |  |  |  | 0.98 (0.82, 1.20) | 0.810 | 1.10 (0.88, 1.40) | 0.440 |
| eGFR (per 1 SD) |  |  |  |  |  | |  |  |  | 0.95 (0.79, 1.10) | 0.580 | 1.40 (1.10, 2.00) | 0.017 |

Model 1: adjusting for sex, age, body mass index, waist-to-hip ratio, smoking, physical activity, alcohol

Model 2: adjusting for sex, age, body mass index, waist-to-hip ratio, smoking, physical activity, alcohol, systolic blood pressure, pulse pressure, hypertention, diabetes mellitus , heart failure, myocardial infarction, stroke, hypoglycaemic drugs

Model 3: adjusting for sex, age, body mass index, waist-to-hip ratio, smoking, physical activity, alcohol, systolic blood pressure, pulse pressure, hypertention, diabetes mellitus , heart failure, myocardial infarction, stroke, hypoglycaemic drugs, fasting blood glucose, creatinine, total cholesterol, high density lipoprotein cholesterol, low density lipoprotein cholesterol, estimating glomerular filtration rate.

| **Supplementary Table 3** Relative Risk of All-cause Mortality in Patients with Stable Coronary Artery Disease by OH 2 | | | | | | | | | | | | |  |
| --- | --- | --- | --- | --- | --- | --- | --- | --- | --- | --- | --- | --- | --- |
| variable | Model 1 | | | | Model 2 | | | | Model 3 | | | |  |
|  | HR(95%CI) | *P*-value | HR(95%CI) | *P*-value | HR(95%CI) | *P*-value | HR(95%CI) | *P*-value | HR(95%CI) | *P*-value | HR(95%CI) | *P*-value | |
|  | **BMI<25** |  | **BMI≥25** |  | **BMI<25** |  | **BMI≥25** |  | **BMI<25** |  | **BMI≥25** |  | |
| OH 2 | 0.98 (0.76, 1.20) | 0.840 | 1.20 (0.94, 1.60) | 0.150 | 0.93 (0.72, 1.20) | 0.580 | 1.20 (0.89, 1.50) | 0.270 | 0.93 (0.72, 1.20) | 0.560 | 1.10 (0.87, 1.50) | 0.360 | |
| Sex | 1.80 (1.10, 2.90) | 0.022 | 2.00 (0.74, 5.50) | 0.170 | 1.60 (0.97, 2.70) | 0.064 | 2.10 (0.76, 5.70) | 0.160 | 1.60 (0.99, 2.70) | 0.057 | 1.70 (0.63, 4.80) | 0.290 | |
| Age | 1.10 (1.10, 1.10) | <0.001 | 1.20 (1.10, 1.20) | <0.001 | 1.10 (1.10, 1.10) | <0.001 | 1.10 (1.10, 1.20) | <0.001 | 1.10 (1.10, 1.10) | <0.001 | 1.20 (1.10, 1.20) | <0.001 | |
| BMI (per 1 SD) | 0.79 (0.65, 0.96) | 0.016 | 1.00 (0.88, 1.30) | 0.600 | 0.78 (0.64, 0.95) | 0.012 | 1.00 (0.82, 1.20) | 0.980 | 0.78 (0.64, 0.96) | 0.017 | 0.99 (0.82, 1.20) | 0.880 | |
| WHR (per 1 SD) | 1.10 (0.97, 1.30) | 0.120 | 1.20 (1.10, 1.40) | 0.001 | 1.10 (0.94, 1.20) | 0.310 | 1.30 (1.10, 1.40) | 0.000 | 1.00 (0.91, 1.20) | 0.500 | 1.30 (1.10, 1.50) | 0.000 | |
| Smoking | 1.20 (0.88, 1.60) | 0.290 | 1.40 (1.00, 2.00) | 0.029 | 1.10 (0.86, 1.50) | 0.350 | 1.30 (0.90, 1.80) | 0.170 | 1.20 (0.86, 1.60) | 0.320 | 1.30 (0.92, 1.80) | 0.140 | |
| Physical activity | 0.56 (0.44, 0.71) | <0.001 | 0.72 (0.53, 0.97) | 0.029 | 0.58 (0.44, 0.75) | <0.001 | 0.71 (0.52, 0.96) | 0.026 | 0.58 (0.44, 0.75) | <0.001 | 0.72 (0.53, 0.99) | 0.043 | |
| some | - | - | - | - | - | - | - | - | - | - | - | - | |
| fairly | 0.51 (0.38, 0.67) | <0.001 | 0.60 (0.43, 0.86) | 0.005 | 0.53 (0.39, 0.71) | <0.001 | 0.58 (0.40, 0.84) | 0.004 | 0.53 (0.39, 0.71) | <0.001 | 0.59 (0.40, 0.85) | 0.005 | |
| quite | 0.37 (0.22, 0.63) | <0.001 | 0.64 (0.35, 1.20) | 0.140 | 0.39 (0.22, 0.68) | 0.001 | 0.61 (0.33, 1.10) | 0.110 | 0.39 (0.22, 0.69) | 0.001 | 0.66 (0.36, 1.20) | 0.180 | |
| Alcohol | 1.20 (0.90, 1.60) | 0.220 | 1.30 (0.94, 1.80) | 0.110 | 1.20 (0.92, 1.70) | 0.170 | 1.40 (1.00, 1.90) | 0.043 | 1.30 (0.93, 1.70) | 0.140 | 1.40 (0.99, 1.90) | 0.057 | |
| SBP (per 1 SD) |  |  |  |  | 1.20 (0.99, 1.40) | 0.070 | 1.00 (0.86, 1.30) | 0.680 | 1.20 (0.97, 1.40) | 0.100 | 1.00 (0.85, 1.20) | 0.830 | |
| PP (per 1 SD) |  |  |  |  | 0.99 (0.83, 1.20) | 0.920 | 0.91 (0.76, 1.10) | 0.340 | 0.99 (0.83, 1.20) | 0.950 | 0.91 (0.76, 1.10) | 0.330 | |
| Hypertention |  |  |  |  | 0.85 (0.64, 1.10) | 0.240 | 1.10 (0.81, 1.50) | 0.540 | 0.82 (0.62, 1.10) | 0.170 | 1.10 (0.79, 1.50) | 0.630 | |
| DM |  |  |  |  | 1.10 (0.82, 1.60) | 0.420 | 1.00 (0.70, 1.50) | 0.880 | 1.10 (0.79, 1.60) | 0.530 | 1.00 (0.71, 1.50) | 0.850 | |
| HF |  |  |  |  | 1.50 (1.10, 2.10) | 0.004 | 2.00 (1.50, 2.80) | <0.001 | 1.60 (1.10, 2.10) | 0.005 | 1.90 (1.40, 2.60) | 0.000 | |
| MI |  |  |  |  | 1.10 (0.85, 1.50) | 0.400 | 1.70 (1.20, 2.30) | 0.001 | 1.10 (0.86, 1.50) | 0.350 | 1.60 (1.20, 2.20) | 0.002 | |
| Stroke |  |  |  |  | 1.40 (1.10, 1.80) | 0.007 | 1.20 (0.87, 1.60) | 0.300 | 1.50 (1.10, 1.90) | 0.004 | 1.10 (0.84, 1.50) | 0.410 | |
| Hypoglycaemic drugs |  |  |  |  | 1.40 (0.93, 2.00) | 0.110 | 1.10 (0.77, 1.70) | 0.500 | 1.30 (0.89, 1.90) | 0.180 | 1.10 (0.71, 1.60) | 0.730 | |
| FBG (per 1 SD) |  |  |  |  |  |  |  |  | 1.10 (0.93, 1.20) | 0.360 | 1.00 (0.90, 1.20) | 0.710 | |
| Creatinine (per 1 SD) |  |  |  |  |  |  |  |  | 1.10 (0.96, 1.20) | 0.250 | 1.80 (1.40, 2.30) | <0.001 | |
| TCHO (per 1 SD) |  |  |  |  |  |  |  |  | 1.00 (0.85, 1.20) | 0.850 | 0.96 (0.76, 1.20) | 0.700 | |
| HDLC (per 1 SD) |  |  |  |  |  |  |  |  | 0.96 (0.84, 1.10) | 0.510 | 0.97 (0.83, 1.10) | 0.710 | |
| LDLC (per 1 SD) |  |  |  |  |  |  |  |  | 0.98 (0.82, 1.20) | 0.820 | 1.10 (0.88, 1.40) | 0.430 | |
| eGFRC |  |  |  |  |  |  |  |  | 0.95 (0.79, 1.10) | 0.560 | 1.40 (1.10, 2.00) | 0.018 | |

Model 1: adjusting for sex, age, body mass index, waist-to-hip ratio, smoking, physical activity, alcohol

Model 2: adjusting for sex, age, body mass index, waist-to-hip ratio, smoking, physical activity, alcohol, systolic blood pressure, pulse pressure, hypertention, diabetes mellitus , heart failure, myocardial infarction, stroke, hypoglycaemic drugs

Model 3: adjusting for sex, age, body mass index, waist-to-hip ratio, smoking, physical activity, alcohol, systolic blood pressure, pulse pressure, hypertention, diabetes mellitus , heart failure, myocardial infarction, stroke, hypoglycaemic drugs, fasting blood glucose, creatinine, total cholesterol, high density lipoprotein cholesterol, low density lipoprotein cholesterol, estimating glomerular filtration rate.

| **Supplementary Table 4** Relative Risk of Cardiovascular disease mortality in Patients with Stable Coronary Artery Disease by OH | | | | | | |
| --- | --- | --- | --- | --- | --- | --- |
| variable | Model 1 | | Model 2 | | Model 3 | |
|  | HR(95%CI) | P-value | HR(95%CI) | P-value | HR(95%CI) | P-value |
| OH | 2.20 (1.50, 3.10) | <0.001 | 1.80 (1.30, 2.60) | 0.001 | 1.90 (1.30, 2.70) | 0.001 |
| Age | 1.20 (1.10, 1.20) | <0.001 | 1.10 (1.10, 1.20) | <0.001 | 1.10 (1.10, 1.20) | <0.001 |
| Smoking | 1.70 (1.10, 2.60) | 0.018 | 1.50 (1.00, 2.40) | 0.051 | 1.40 (0.89, 2.20) | 0.140 |
| Alcohol | 1.70 (1.10, 2.50) | 0.018 | 1.50 (0.97, 2.20) | 0.070 | 1.60 (1.10, 2.50) | 0.022 |
| SBP (per 1 SD) |  |  | 1.00 (0.99, 1.00) | 0.180 | 1.10 (0.85, 1.40) | 0.510 |
| PP (per 1 SD) |  |  | 1.00 (0.99, 1.00) | 0.430 | 1.20 (0.89, 1.50) | 0.260 |
| Hypertention |  |  | 1.10 (0.67, 1.70) | 0.790 | 1.00 (0.64, 1.70) | 0.910 |
| DM |  |  | 1.40 (0.94, 1.90) | 0.099 | 1.60 (0.97, 2.60) | 0.066 |
| HF |  |  | 3.00 (2.00, 4.40) | <0.001 | 2.90 (1.90, 4.20) | 0.000 |
| MI |  |  | 3.20 (2.20, 4.60) | <0.001 | 3.10 (2.20, 4.50) | 0.000 |
| Hypoglycaemic drugs |  |  | 0.83 (0.49, 1.40) | 0.480 | 0.70 (0.41, 1.20) | 0.200 |
| Creatinine (per 1 SD) |  |  |  |  | 1.10 (0.88, 1.30) | 0.590 |
| TCHO (per 1 SD) |  |  |  |  | 0.71 (0.57, 0.89) | 0.003 |
| HDLC (per 1 SD) |  |  |  |  | 0.89 (0.71, 1.10) | 0.310 |
| LDLC (per 1 SD) |  |  |  |  | 1.60 (1.30, 1.90) | 0.000 |
| eGFR (per 1 SD) |  |  |  |  | 0.95 (0.66, 1.40) | 0.770 |

Model 1: adjusting for age, smoking, alcohol

Model 2: adjusting for age, smoking, alcohol, systolic blood pressure, pulse pressure, hypertention, diabetes mellitus , heart failure, myocardial infarction, hypoglycaemic drugs

Model 3: adjusting for age, smoking, alcohol, systolic blood pressure, pulse pressure, hypertention, diabetes mellitus, heart failure, myocardial infarction, hypoglycaemic drugs, creatinine, total cholesterol, high density lipoprotein cholesterol, low density lipoprotein cholesterol, estimating glomerular filtration rate.

| **Supplementary Table 5** Relative Risk of Cardiovascular disease mortality in Patients with Stable Coronary Artery Disease by OH 0 | | | | | | | |
| --- | --- | --- | --- | --- | --- | --- | --- |
| variable | Model 1 | | Model 2 | | Model 3 | | |
|  | HR(95%CI) | P-value | HR(95%CI) | P-value | HR(95%CI) | | P-value |
| OH 0 | 1.70 (1.20, 2.40) | 0.005 | 1.50 (1.00, 2.10) | 0.028 | 1.50 (1.00, 2.10) | | 0.037 |
| Age | 1.20 (1.10, 1.20) | <0.001 | 1.10 (1.10, 1.20) | <0.001 | 1.10 (1.10, 1.20) | | <0.001 |
| Smoking | 1.70 (1.10, 2.50) | 0.025 | 1.50 (0.98, 2.30) | 0.064 | 1.30 (0.86, 2.10) | | 0.190 |
| Alcohol | 1.70 (1.10, 2.60) | 0.013 | 1.50 (1.00, 2.30) | 0.048 | 1.60 (1.10, 2.50) | | 0.025 |
| SBP (per 1 SD) |  |  | 1.20 (0.93, 1.50) | 0.170 | 1.10 (0.85, 1.40) | | 0.490 |
| PP (per 1 SD) |  |  | 1.10 (0.85, 1.40) | 0.440 | 1.20 (0.89, 1.50) | | 0.280 |
| Hypertention |  |  | 1.10 (0.69, 1.80) | 0.680 | 1.00 (0.65, 1.70) | | 0.860 |
| DM |  |  | 1.60 (0.98, 2.60) | 0.063 | 1.60 (1.00, 2.70) | | 0.052 |
| HF |  |  | 3.10 (2.10, 4.60) | <0.001 | 3.10 (2.10, 4.60) | | <0.001 |
| MI |  |  | 3.20 (2.20, 4.60) | <0.001 | 3.10 (2.10, 4.50) | | <0.001 |
| Antihyperglycemic drug |  |  | 0.82 (0.49, 1.40) | 0.450 | 0.68 (0.39, 1.20) | | 0.160 |
| Creatinine (per 1 SD) |  |  |  |  | 1.00 (0.86, 1.20) | | 0.760 |
| TCHO (per 1 SD) |  |  |  |  | 0.72 (0.58, 0.90) | | 0.004 |
| HDLC (per 1 SD) |  |  |  |  | 0.88 (0.70, 1.10) | | 0.260 |
| LDLC (per 1 SD) |  |  |  |  | 1.50 (1.30, 1.90) | | <0.001 |
| eGFR (per 1 SD) |  |  |  |  | | 0.92 (0.63, 1.30) | 0.660 |

Model 1: adjusting for age, smoking, alcohol

Model 2: adjusting for age, smoking, alcohol, systolic blood pressure, pulse pressure, hypertention, diabetes mellitus , heart failure, myocardial infarction, hypoglycaemic drugs

Model 3: adjusting for age, smoking, alcohol, systolic blood pressure, pulse pressure, hypertention, diabetes mellitus, heart failure, myocardial infarction, hypoglycaemic drugs, creatinine, total cholesterol, high density lipoprotein cholesterol, low density lipoprotein cholesterol, estimating glomerular filtration rate.

| **Supplementary Table 6** Relative Risk of Cardiovascular disease mortality in Patients with Stable Coronary Artery Disease by OH 2 | | | | | | |
| --- | --- | --- | --- | --- | --- | --- |
| variable | Model 1 | | Model 2 | | Model 3 | |
|  | HR(95%CI) | P-value | HR(95%CI) | P-value | HR(95%CI) | P-value |
| OH 2 | 1.70 (1.20, 2.50) | 0.006 | 1.60 (1.10, 2.40) | 0.009 | 1.50 (1.00, 2.20) | 0.034 |
| Age | 1.20 (1.10, 1.20) | <0.001 | 1.10 (1.10, 1.20) | <0.001 | 1.10 (1.10, 1.20) | <0.001 |
| Smoking | 1.70 (1.10, 2.60) | 0.016 | 1.50 (0.99, 2.30) | 0.057 | 1.40 (0.87, 2.10) | 0.180 |
| Alcohol | 1.70 (1.10, 2.60) | 0.010 | 1.60 (1.00, 2.40) | 0.035 | 1.70 (1.10, 2.50) | 0.020 |
| SBP (per 1 SD) |  |  | 1.20 (0.94, 1.60) | 0.140 | 1.10 (0.86, 1.40) | 0.450 |
| PP (per 1 SD) |  |  | 1.10 (0.84, 1.40) | 0.520 | 1.10 (0.88, 1.50) | 0.320 |
| Hypertention |  |  | 1.10 (0.71, 1.80) | 0.590 | 1.10 (0.66, 1.70) | 0.790 |
| DM |  |  | 1.60 (0.98, 2.60) | 0.063 | 1.60 (1.00, 2.70) | 0.048 |
| HF |  |  | 3.20 (2.10, 4.70) | <0.001 | 3.10 (2.10, 4.60) | <0.001 |
| MI |  |  | 3.20 (2.20, 4.70) | <0.001 | 3.20 (2.20, 4.60) | <0.001 |
| Antihyperglycemic drug |  |  | 0.84 (0.50, 1.40) | 0.520 | 0.69 (0.40, 1.20) | 0.190 |
| Creatinine (per 1 SD) |  |  |  |  | 1.00 (0.87, 1.30) | 0.640 |
| TCHO (per 1 SD) |  |  |  |  | 0.74 (0.59, 0.93) | 0.008 |
| HDLC (per 1 SD) |  |  |  |  | 0.88 (0.70, 1.10) | 0.260 |
| LDLC (per 1 SD) |  |  |  |  | 1.50 (1.20, 1.80) | <0.001 |
| eGFR (per 1 SD) |  |  |  |  | 0.93 (0.63, 1.40) | 0.700 |

Model 1: adjusting for age, smoking, alcohol

Model 2: adjusting for age, smoking, alcohol, systolic blood pressure, pulse pressure, hypertention, diabetes mellitus , heart failure, myocardial infarction, Antihyperglycemic drug

Model 3: adjusting for age, smoking, alcohol, systolic blood pressure, pulse pressure, hypertention, diabetes mellitus, heart failure, myocardial infarction, Antihyperglycemic drug, creatinine, total cholesterol, high density lipoprotein cholesterol, low density lipoprotein cholesterol, estimating glomerular filtration rate.

| **Supplementary Table 7**  Cause-specific risk regression modelling results | | |
| --- | --- | --- |
|  | sHR(95%CI) | *P*-value |
| Cardiovascular Disease Death Outcome |  |  |
| Subject with OH | 1.74[1.22, 2.49] | 0.002 |
| Competitive Risk Incident Death Outcome |  |  |
| Subject with OH | 1.04 [0.84, 1.27] | P=0.738 |

| **Supplementary Table 8** Univariate and multivariate competition risk modelling for Cardiovascular Disease Mortality | | | | |
| --- | --- | --- | --- | --- |
| variable | Univariate analysis | | Multivariate analysis | |
|  | sHR(95%CI) | *P*-value | sHR(95%CI) | *P*-value |
| OH | 2.31 (1.63, 3.28) | <0.001 | 1.91 (1.30, 2.70) | <0.001 |
| Age | 1.11 (1.06, 1.15) | <0.001 | 1.10 (1.00, 1.20) | <0.001 |
| Smoking | 1.07 (0.66, 1.72) | 0.780 | - | - |
| Alcohol | 1.59 (1.05, 2.40) | 0.027 | 1.54 (1.00, 2.40) | 0.049 |
| SBP (per 1 SD) | 1.32 (1.12, 1.57) | 0.001 | 1.09 (0.85, 1.40) | 0.510 |
| PP (per 1 SD) | 1.37 (1.16, 1.62) | <0.001 | 1.18 (0.91, 1.50) | 0.220 |
| Hypertention | 1.54 (0.98, 2.42) | 0.062 | - | - |
| DM | 1.58 (1.12, 2.24) | 0.010 | 1.64 (1.00, 2.70) | 0.050 |
| HF | 4.41 (3.09, 6.29) | <0.001 | 2.59 (1.70, 3.90) | <0.001 |
| MI | 3.97 (2.79, 5.64) | <0.001 | 2.98 (2.00, 4.40) | <0.001 |
| Antihyperglycemic drug | 1.46 (1.01, 2.10) | 0.044 | 0.62 (0.36, 1.10) | 0.076 |
| Creatinine (per 1 SD) | 1.13 (0.99, 1.29) | 0.080 | - | - |
| TCHO (per 1 SD) | 0.82 (0.67, 0.99) | 0.046 | 0.63 (0.51, 0.79) | <0.001 |
| HDLC (per 1 SD) | 0.77 (0.63, 0.94) | 0.009 | 0.92 (0.73, 1.20) | 0.490 |
| LDLC (per 1 SD) | 1.30 (1.11, 1.53) | 0.002 | 1.82 (1.50, 2.20) | <0.001 |
| eGFR (per 1 SD) | 0.77 (0.58, 1.03) | 0.075 | - | - |
